# The 2-methylcitrate cycle and the glyoxylate shunt in *Pseudomonas aeruginosa* are linked through enzymatic redundancy

**DOI:** 10.1016/j.jbc.2025.108355

**Published:** 2025-02-25

**Authors:** Andre J. Wijaya, Stephen K. Dolan, Michael Kohlstedt, Lars Gläser, Paul Brear, Stephen Geddis, Christoph Wittmann, David R. Spring, Martin Welch

**Affiliations:** 1 Department of Biochemistry, University of Cambridge, Cambridge, United Kingdom; 2 Department of Genetics and Biochemistry, Eukaryotic Pathogens Innovation Center, Clemson University, Clemson, USA; 3 Institute of Systems Biotechnology, Saarland University, Saarbrücken, Germany; 4 Department of Chemistry, University of Cambridge, Cambridge, United Kingdom

**Keywords:** *Pseudomonas aeruginosa*, enzyme promiscuity, glyoxylate shunt, 2-methylcitrate cycle, 2-methylisocitrate lyase, isocitrate lyase

## Abstract

The 2-methylcitrate cycle and the glyoxylate cycle are central metabolic pathways in *Pseudomonas aeruginosa*, enabling the organism to utilize organic acids such as propionate and acetate during infection. Here, we show that these cycles are linked through enzymatic redundancy, with isocitrate lyase (AceA) exhibiting secondary 2-methylisocitrate lyase activity. Furthermore, we use a combination of structural analyses, enzyme kinetics, metabolomics, and targeted mutation of PrpB_Pa_ to demonstrate that whereas loss of PrpB function impairs growth on propionate, the promiscuous 2-methylisocitrate lyase activity of AceA compensates for this by mitigating the accumulation of toxic 2-methylcitrate cycle intermediates. Our findings suggest that simultaneous inhibition of PrpB and AceA could present a robust antimicrobial strategy to target *P. aeruginosa* in propionate-rich environments, such as the cystic fibrosis airways. Our results emphasize the importance of understanding pathway interconnections in the development of novel antimicrobial agents.

Short-chain fatty acids such as propionate are abundant in certain infection scenarios, and readily accumulate to mM concentrations (1). The metabolism of short-chain fatty acids is becoming increasingly topical, especially given their potent immune modulatory activity (2). The 2-methylcitrate cycle (2-MCC) is one of the main metabolic pathways used by bacteria to metabolize propionate. The 2-MCC can also be regarded as a propionate detoxification pathway (3, 4, 5). The enzyme 2-methylisocitrate lyase (2-MICL, PrpB) catalyzes the last step of the 2-MCC. Its function is to cleave the toxic intermediate, 2-methylisocitrate (2-MIC), into pyruvate and succinate, enabling these metabolic precursors to feed directly into central metabolism (Fig. 1*A*). This step has been suggested as a potential point of antimicrobial intervention for several microorganisms (6, 7, 8).

**Figure 1:**
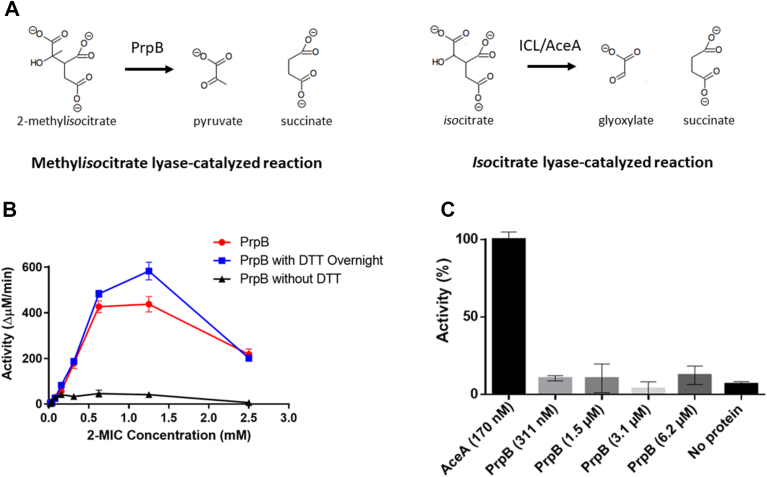
**Biochemical characterization of recombinant PrpB_Pa_.***A*, schematic of the reactions catalyzed by PrpB_Pa_ and AceA (ICL_Pa_). [Note that, as shown later in this report, ICL_Pa_ also catalyzes the methylisocitrate lyase reaction.] *B*, shows a plot of reaction velocity *versus* substrate (2-methylisocitrate, 2-MIC) concentration in samples containing untreated PrpB_Pa_ (*black triangles*) or PrpB_Pa_ pretreated with 5 mM DTT for 10 min (*red circles*). *Blue squares* show the kinetic behavior of PrpB_Pa_ that had been pretreated overnight with DTT. *C*, isocitrate lyase activity of PrpB_Pa_ and ICL_Pa_ (as indicated) measured using a phenylhydrazine-based assay. ICL_Pa_ (1 μg, 170 nM) could catalyze the cleavage of isocitrate into glyoxylate and succinate, whereas no detectable isocitrate lyase activity was associated with PrpB_Pa_ at any of the concentrations tested (ranging from 311 nM to 6.2 μM, as indicated). For (*B* and *C*), the data are representative of two independent experiments, each performed in triplicate. Error bars represent standard deviation of the mean. ICL, isocitrate lyase.

The prominent Gram-negative human pathogen, *Pseudomonas aeruginosa* utilizes organic acids such as propionate as a carbon source during infection (9). We previously showed that the methylcitrate synthase, PrpC, which catalyzes the first step of the 2-MCC, is a possible target for antimicrobial development. However, PrpC has structural features which are shared with citrate synthase from other bacterial and mammalian systems, potentially making targeting more complex (10). In the current work, we characterize the X-ray crystal structure of the final enzyme in the 2-MCC, PrpB (2-MICL) from *P. aeruginosa* (hereafter, PrpB_Pa_). We further use a combination of enzyme kinetic analyses, reverse genetics, and metabolomics to demonstrate how the organism responds when the 2-MICL activity is lost (*e.g.*, due to mutation of *prpB*_Pa_, or due to chemical inhibition of the enzyme). Finally, we show that a *prpB*_Pa_ deletion mutant is partially protected from the toxic impact of 2-MIC accumulation through the secondary activity of *aceA*-encoded isocitrate lyase (ICL), which conventionally functions in the glyoxylate cycle (Fig. 1*A*). This new knowledge into the structural and biochemical properties of PrpB_Pa_ provides valuable insights into how best to target this pathway and block growth of *P. aeruginosa* during infection.

## Results

### Catalytic properties and regulation of *P. aeruginosa* PrpB

To directly assess the enzymatic activity of PrpB_Pa_, we purified this enzyme to investigate its specificity and kinetic properties *in vitro*. The *prpB*_Pa_ gene was cloned and overexpressed (with a cleavable His_6_ tag) in *Escherichia coli* and purified to homogeneity. The purified enzyme was then assayed for *threo*-2-methylisocitrate lyase (2-MICL) activity. Using a lactate dehydrogenase (LDH) coupled assay (11) we determined the kinetic parameters of PrpB_Pa_ for 2-MIC. Here, the pyruvate generated as a result of PrpB_Pa_ activity is converted to lactate by the LDH, with concomitant oxidation of NADH. However, we noted kinetics consistent with substrate inhibition, which we reasoned was due to the LDH coupling reaction since LDH activity is known to be vulnerable to this (12). Hence, the kinetic parameters were calculated assuming substrate inhibition, using GraphPad Prism 6 (https://www.graphpad.com). We found that DTT was essential for optimal activity of PrpB_Pa_ *in vitro*; 10 min preincubation with DTT was sufficient to achieve 95% activation (assuming 100% activity following overnight incubation with the thiol, Fig. 1*B*). Calculated kinetic parameters for PrpB_Pa_ with for 2-MIC as a substrate were K_M_ = 632 ± 158 μM, k_cat_ = 48.2 ± 4.2 s^−1^, and k_cat_/K_M_ = 7.6 × 10^4^ M^−1^ s^−1^. The PrpB enzymes from *E. coli*, *Salmonella enterica* serovar Typhimurium, and *Aspergillus nidulans* have uniformly lower K_M_ values (19 μM, 19 μM, and 31 μM, respectively) than PrpB_Pa_, but variable k_cat_ values (12 s^−1^, 74 s^−1^, and 198 s^−1^, respectively). We do not know the reason for these disparities, since in most other respects (activation by thiols, structural similarity, behavior in solution and so on), PrpB_Pa_ appears very similar to PrpB from these other species.

We next examined whether PrpB_Pa_ might be inhibited by common central metabolic intermediates or other low-molecular weight compounds. This is relevant because the analogous glyoxylate shunt enzyme (ICL_Pa_) is subject to robust allosteric regulation (13). To do this, we screened the activity of PrpB_Pa_ against a panel of metabolites (Fig. S1). [Note that, due to their interaction with LDH, a number of potential regulators could not be tested using this assay, including NADH, NADPH, phosphoenolpyruvate, pyruvate, ATP, and ADP]. Of the potential physiological regulators that we tested, α-*keto*glutarate, *cis*-aconitic acid, and D-glyceraldehyde 3-phosphate were all potent inhibitors of PrpB_Pa_ (Fig. S1). Maleic acid was a nonphysiological inhibitor. We also found, in agreement with the literature, that 3-nitropropionic acid was also a potent inhibitor of PrpB_Pa_ (P < 1 × 10^−4^) (14).

The 2-MCC methylcitrate synthase (PrpC) from *P. aeruginosa* catalyzes the condensation of oxaloacetate and propionyl-CoA. In a parallel reaction, the TCA cycle enzyme, citrate synthase (GltA), catalyzes the condensation of oxaloacetate and acetyl-CoA. We recently showed that PrpC_Pa_ also possesses robust secondary citrate synthase activity (10). Since methylisocitrate lyase and ICL both catalyze biochemically similar steps in the 2-methylcitrate and glyoxylate cycles, respectively, this made us wonder whether PrpB_Pa_ might also catalyze the ICL reaction and cleave isocitrate to yield succinate and glyoxylate. However, even at the highest concentration of isocitrate (500 μM) and PrpB_Pa_ (6.2 μM) tested, we could not detect any glyoxylate formation arising from the cleavage of isocitrate (Fig. 1*C*), even following incubation of the reaction mixtures overnight (data not shown). We conclude that PrpB_Pa_ does not possess ICL activity. We note that PrpB from *E. coli* and *Aspergillus fumigatus* also shows similar specificity for methylisocitrate over *iso*citrate (11, 15).

### Structure of PrpB_Pa_

To gain insights into the possible structural bases for these kinetic data, we used X-ray crystallography to solve the structure of PrpB_Pa_ in both the apo-form, and in the presence of bound Mg^2+^/pyruvate. The pyruvate was introduced by crystal soaking in the presence of an equimolar (30 mM each) solution of pyruvate and succinate. Mg^2+^ was not included in the soak, or in the crystallization buffer, so was presumably acquired as a contaminant in these buffers, or was present in the purified enzyme preparations. The crystal structure of apo-PrpB_Pa_ was solved by molecular replacement (MR) method using the structure of *E. coli* PrpB as the search template (Protein Data Bank (PDB) ID: 1MUM). For the Mg^2+^/pyruvate bound PrpB_Pa_, apo- PrpB_Pa_ was used as the MR template. The structures were solved to 1.8 Å for apo-PrpB_Pa_ and 1.76 Å for holo-PrpB_Pa_.

**Figure 2:**
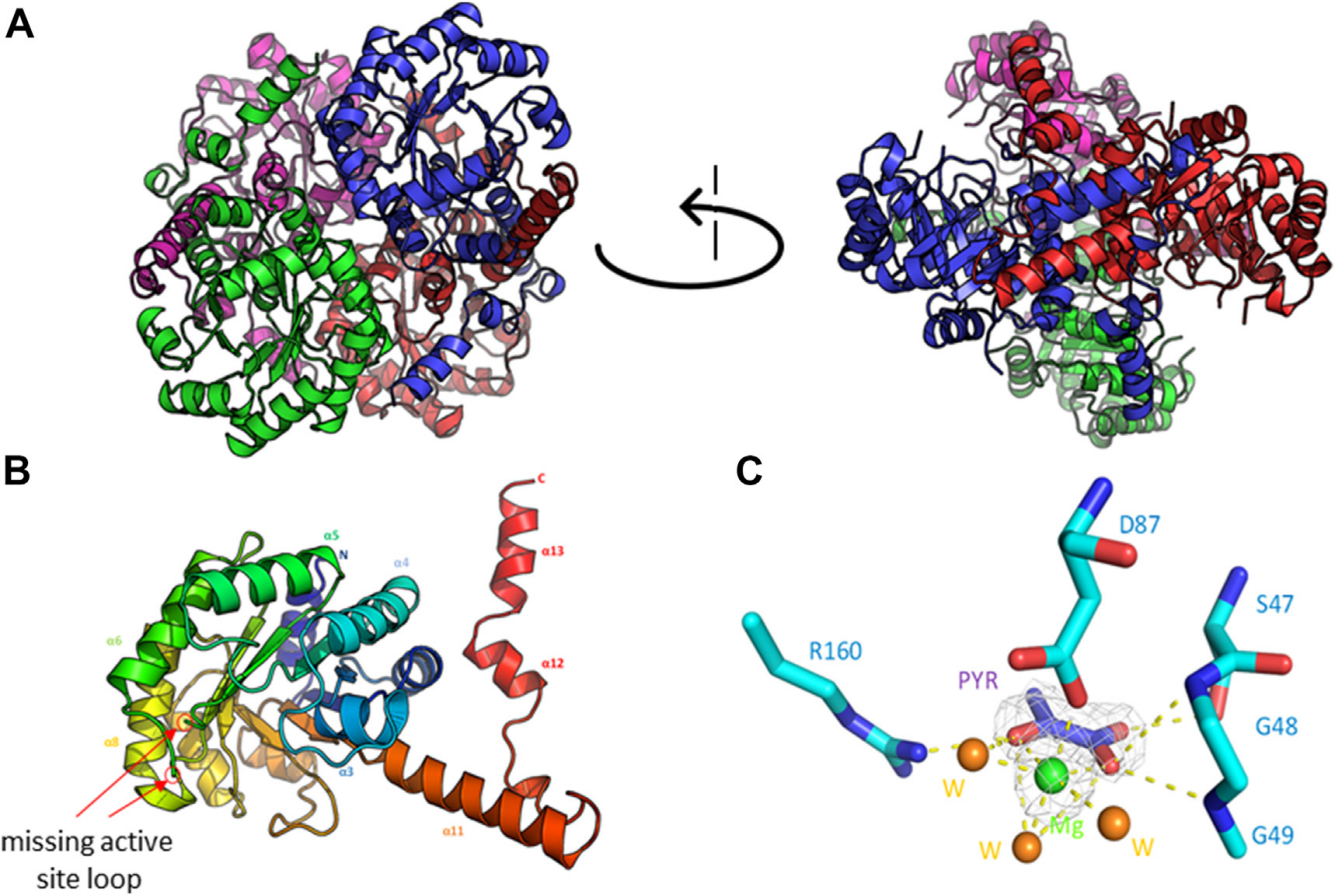
**Crystal structure of PrpB_Pa_.***A*, shows the homotetramer (dimer of dimers) quaternary structure from PrpB_Pa_. The protomers are colored *red* (protomer A), *blue* (protomer B), *green* (protomer C), and *magenta* (protomer D). Note the swapping of α11 from protomers A and B. *B*, monomer of apo-PrpB_Pa_ colored *rainbow* (*blue* to *red*) from the N to C terminal. The missing active site loop (residue 121–131) is *circled* in *red* and indicated with *red arrows*. *C*, close-up view around the Mg^2+^/pyruvate binding site. The Mg^2+^ (*green sphere*) is hexahedrally coordinated by three water molecules, two oxygen atoms from pyruvate, and the carboxyl group of D87. The pyruvate (PYR, *deep blue*) and amino acid (*cyan*) residues are shown as *sticks*. Water molecules (W) are shown as *orange spheres*. Polar interactions are shown with a *dashed line*. The electron density map (2*F*_*o*_*-F*_*c*_) around the Mg^2+^ and pyruvate is shown in *white* and is contoured at 1.5σ.

In the asymmetric unit, like all the characterized PrpB and ICL structures to date, PrpB_Pa_ formed a tetrameric (dimer of dimers) quaternary structure (13, 16, 17) (Fig. 2*A*). This also agrees with our analytical ultracentrifugation data which suggest a tetrameric assembly of PrpB_Pa_ in solution (Fig. S2). All four protomers in each of the apo-and holo-structures had an almost identical conformation (RMSD < 0.18 Å). Additionally, no significant changes were seen in protomer conformation when comparing the apo- and holo-PrpB_Pa_ structures (RMSD 0.19 Å). In both structures, the electron density for most of the amino acid residues was clearly resolved. However, electron density for the first and last seven amino acid residues at the C and N termini, and 11 residues around the active site loop (residues 121–131, circled in Fig. 2*B*) were missing. Even with the product and cofactor bound (Mg^2+^/pyruvate), the active site loop of PrpB_Pa_ remained unresolved, suggesting that this loop is intrinsically flexible in these conditions. This was also reported for the *S. enterica* serovar Typhimurium PrpB structure (18). The overall model stereochemistry in the Ramachandran plot (19) showed that 99.6% of the amino acids were within the preferred/allowed regions.

PrpB_Pa_ belongs to the pyruvate/phosphoenolpyruvate kinase-like superfamily (IPR015813). All members of this family, except the ketopantoate hydroxymethyl transferase, contain a central triose phosphate isomerase-barrel fold with an extensive dimer interface involving helix swapping. Figure 2*A* highlights an example of this helix swapping involving α11 on protomers A and B in the PrpB_Pa_ structure.

Several structures of PrpB homologs in both apo- and holo-form from *E. coli*, *S. enterica* serovar Typhimurium, and *Burkholderia pseudomallei* have been solved PDB (*E. coli* PDB ID: 1MUM, 1OQF, 1XG3, 1XG4; *S. enterica* PDB ID: 1O5Q, 1UJQ; *B. pseudomallei* PDB ID: 3EOO). The amino acid sequences and structures of all these PrpB homologs are similar to that of PrpB_Pa_ (sequence identity >65%, Cα RMSD <1.5 Å). Unsurprisingly, the catalytic site residues are conserved; the detailed catalytic mechanism of PrpB has been proposed previously by Liu *et al.* (17). Although not resolved in the structure(s) here, PrpB_Pa_ contains the canonical PrpB motif (KRCGH, residues 123–128, highlighted in Fig. 2*B*) (20). Multiple conformations of this catalytic loop region have been reported previously (16, 17). We note that this loop region is slightly different from the analogous one in ICL enzymes, which contain a conserved KKCGH motif (13, 18).

The active site of the ICL superfamily (which includes PrpB) contains an essential Mg^2+^, which is crucial for enzymatic activity. In our holo-PrpB_Pa_ structure, the Mg^2+^ and pyruvate could be modeled with confidence (Fig. 2*C*). Mg^2+^ was bound in a negatively charged pocket comprised by the side chains of D60, D87, D89, and E117. The Mg^2+^ was hexahedrally coordinated by three water molecules, two oxygen atoms from the pyruvate, and by the side chain of D87 (Fig. 2*C*). The binding of Mg^2+^ in PrpB_Pa_ did not change the Cα geometry of D87. This contrasts with the situation in *E. coli* PrpB, where Mg^2+^ binding has been reported to shift the *ɸ* and ψ angle of D87 into an unfavorable region of the Ramachandran plot (19).

### Structural comparison of *P. aeruginosa* PrpB and ICL

**Figure 3:**
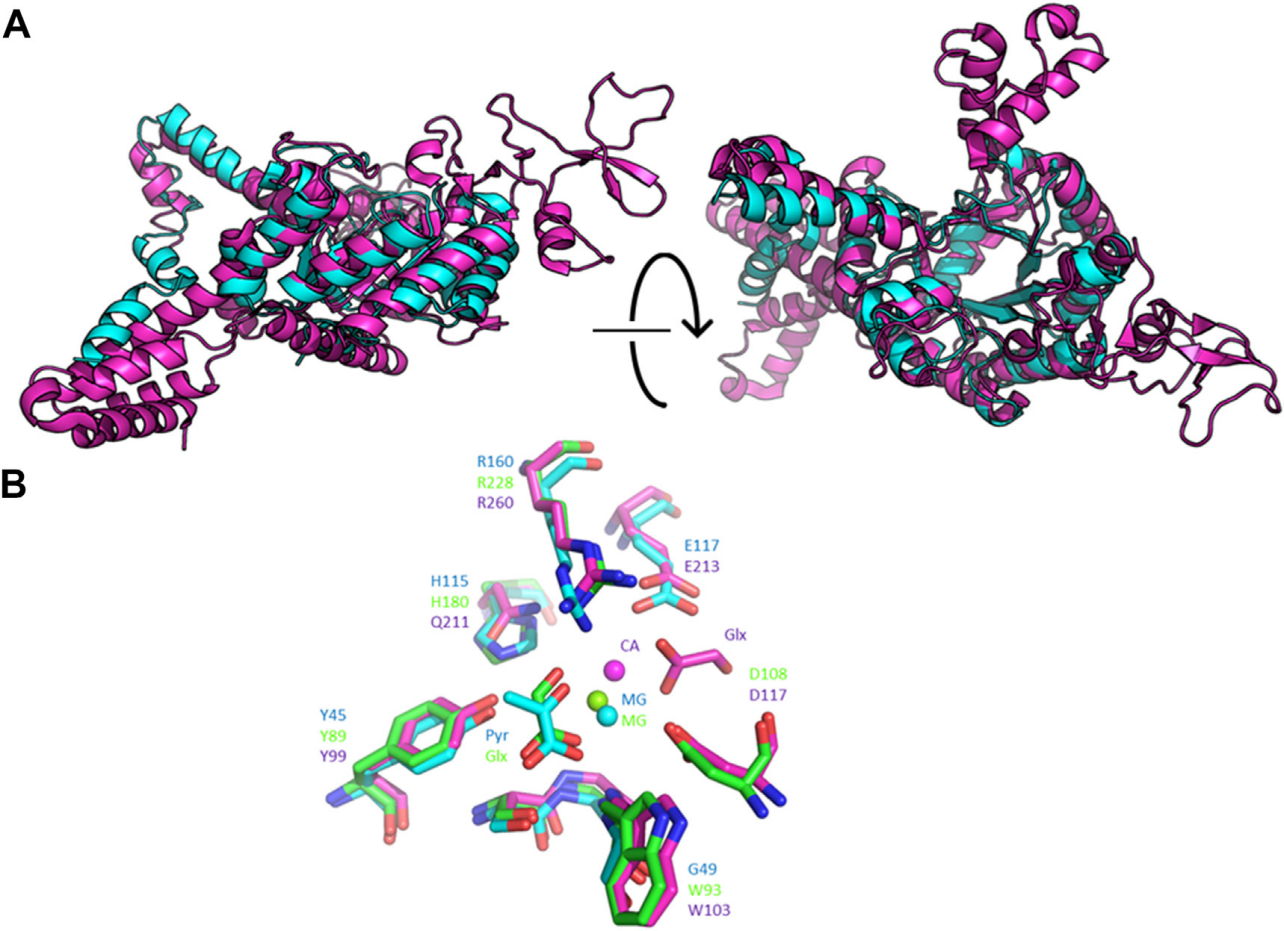
**The tertiary structures of PrpB_Pa_ and AceA_Pa_ are conserved.***A*, superimposed secondary structures of PrpB_Pa_ (*cyan*) and AceA_Pa_ (*magenta*, PDB 6G1O). Note that the secondary structures of the proteins are very similar, even though the sequence identity is relatively low (36%). *B*, pyruvate (Pyr)/glyoxylate (Glx) binding site of PrpB_Pa_ (*cyan*), AceA_Pa_ (*magenta*) and AceA_Mtb_ (*green*, PDB no. 1F8I). Some of the active site residues are conserved (Y45, R160; PrpB_Pa_ numbering). In PrpB_Pa_ and AceA_Mtb_, the binding mode of pyruvate and glyoxylate is identical, whereas in AceA_Pa_ the departing product molecule is displaced. Additionally, PrpB_Pa_ and AceA_Mtb_ contain bound Mg^2+^ (MG), whereas AceA_Pa_ contains a bound Ca^2+^ (CA). PDB, Protein Data Bank.

*P. aeruginosa* ICL is encoded by *aceA*. We previously solved the X-ray crystal structure of AceA_Pa_ (13). This allowed us to compare the tertiary structures of PrpB_Pa_ and AceA_Pa_. The superimposed structures are shown in Figure 3*A*. Despite the relatively low amino acid sequence identity (36%) between PrpB_Pa_ and AceA_Pa_, their core structural folds were very similar. However, AceA_Pa_ is significantly larger (58.9 kDa) than PrpB_Pa_ (32.1 kDa), and AceA_Pa_ contains an additional β-loop-β motif and an α-helical bundle that are absent from the structure of PrpB_Pa_.

The inner core αβ triose phosphate isomerase barrel of PrpB_Pa_ and AceA_Pa_ contains the binding site for pyruvate and glyoxylate (respectively), and some of the residues that interact with these reaction products are conserved between these proteins (Y45, R160; PrpB_Pa_ numbering), as well as in AceA from other organisms such as *Mycobacterium tuberculosis* (Fig. 3*B*). Interestingly, the position occupied by Mg^2+^ in PrpB_Pa_ is occupied by a Ca^2+^ ion in AceA_Pa_. Additionally, we note that the binding mode of pyruvate and glyoxylate in PrpB_Pa_ and in *M. tuberculosis* AceA (AceA_Mtb_), respectively, is identical, whereas in AceA_Pa_, the glyoxylate is rotated by almost 90° and translated by 4.3 Å (Fig. 3*B*). In the PrpB_Pa_ structure, the pyruvate interacts with the enzyme *via* the hydroxyl oxygen atoms of Y45 and S47, the peptide bond nitrogen from G48 and G49, and a guanidino group nitrogen from R160. By contrast, in AceA_Pa_, the glyoxylate interacts with the enzyme *via* the carboxylate side chains of D117, D186, and E213, and *via* the hydroxyl oxygen atom of S217 (PDB: 6G1O). The binding mode of pyruvate and glyoxylate in PrpB_Pa_ and AceA_Pa_, respectively, is therefore distinct.

### A possible dual function for AceA_Pa_

**Figure 4:**
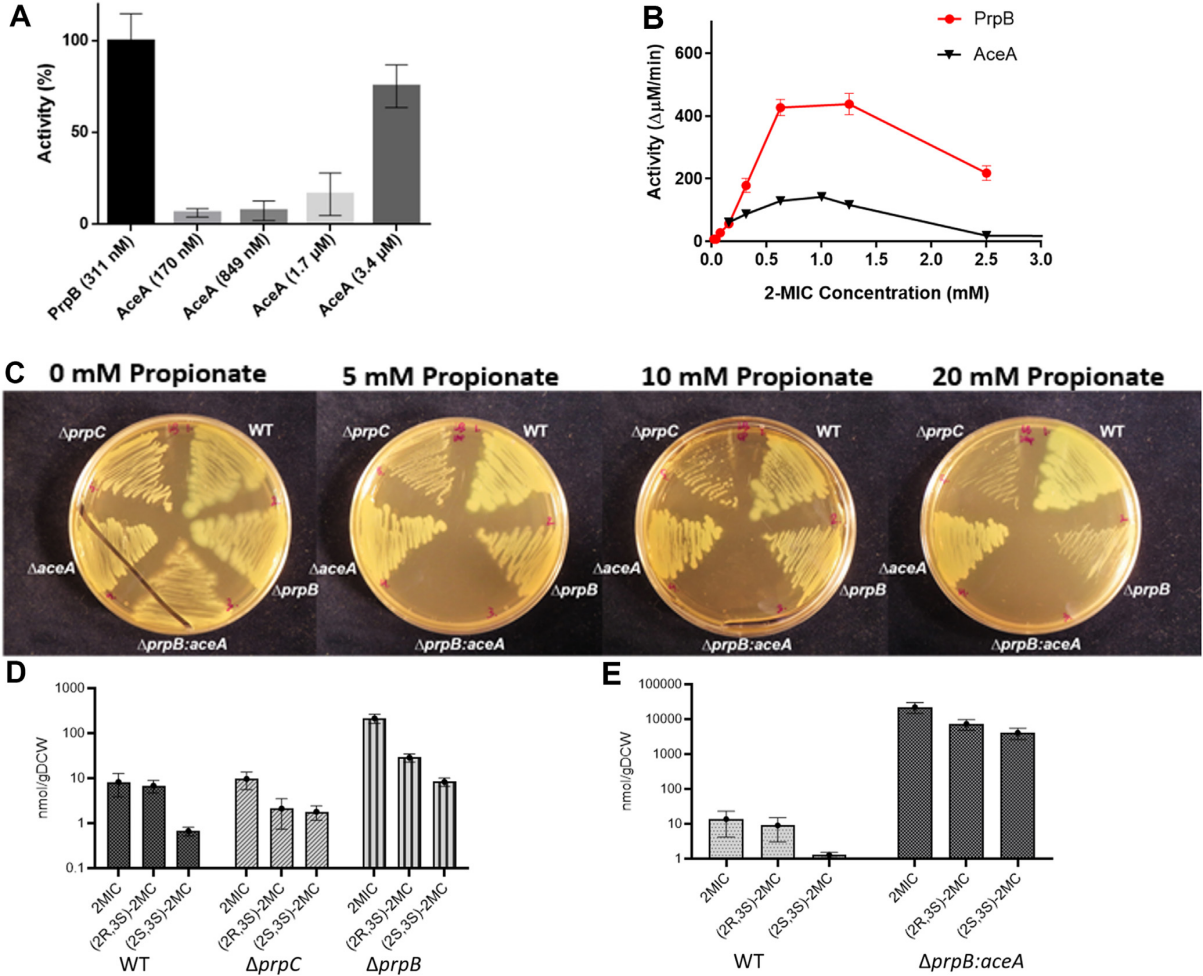
**The secondary 2-methylisocitrate lyase (2-MICL) activity of AceA blunts 2-MIC toxicity in *Pseudomonas aeruginosa*.***A*, relative 2-MICL activity of the indicated concentrations of PrpB_Pa_ and AceA_Pa_ measured using the LDH-coupled reaction (1 mM 2-MIC). Full (100%) activity of PrpB_Pa_ is set at 100%. The data are representative of two independent experiments, each performed in triplicate. Error bars represent standard deviation of the mean. *B*, reaction velocity *versus* substrate (2-methylisocitrate, 2-MIC) concentration. AceA_Pa_ (*blue* symbols and *line*) and PrpB_Pa_ were preincubated with 5 mM DTT prior to assaying. The PrpB_Pa_ data are the same as those shown in Figure 1*A*. The data are representative of two independent experiments, each performed in triplicate. Error bars represent standard deviation of the mean. *C*, growth of the WT and deletion mutants of *P. aeruginosa* on complex medium (LB-Lennox) agar containing increasing amounts of propionate (0–20 mM, as indicated). The data are representative of three independent experiments. *D*, accumulation of intracellular 2-MCC intermediates in the WT (PAO1), a Δ*prpC* mutant and a Δ*prpB* mutant following 3 h exposure to propionate (5 mM). The measured intermediates were (2*R*,3*S*)-2-methylcitrate, (2*S*, 3*S*)-2-methylcitrate, and 2-methylisocitrate. Note the log-scale on the *y*-axis. The data are representative of biological triplicates. Error bars represent standard deviation of the mean. *E*, accumulation of intracellular 2-methylcitrate intermediates in the WT (PAO1), and in a Δ*prpB* Δ*aceA* double mutant following 1 h exposure to propionate (5 mM). The data are representative of biological triplicates. Error bars represent standard deviation of the mean. 2-MCC, 2-methylcitrate cycle; LDH, lactate dehydrogenase.

We have previously noted that the enzymes of the glyoxylate shunt (ICL and malate synthase (GlcB)) are both highly expressed during growth of *P. aeruginosa* on propionate as a sole carbon source (10). This high-level expression of AceA_Pa_ and GlcB_Pa_ is likely due to RccR-mediated derepression of the glyoxylate cycle genes (21). Nevertheless, and in spite of the robust expression of AceA_Pa_ and GlcB_Pa_, very little carbon was fluxed through the glyoxylate shunt during growth on propionate (10). Although PrpB_Pa_ lacks ICL activity (Fig. 1), this observation raised the possibility that AceA_Pa_ might also play a role in the 2-MCC. We therefore decided to evaluate whether AceA_Pa_ could catalyze the cleavage of 2-MIC (the reaction normally carried out by PrpB_Pa_). ICL from other bacterial and fungal sources have been previously shown to exhibit some 2-MICL activity, although to our knowledge, the functional significance (if any) of this potential pathway crosstalk has not been investigated before. Although its activity was lower than that of PrpB_Pa_, purified AceA_Pa_ demonstrated robust 2-MICL activity *in vitro* (Fig. 4, *A* and *B*). Using the LDH-coupled reaction, the specificity constant (k_cat_/K_M_) of AceA_Pa_ for 2-MIC was calculated to be 3.4 × 10^3^ M^−1^ s^−1^ (*cf.* 7.6 × 10^4^ M^−1^ s^−1^ for PrpB_Pa_). This clear secondary activity suggested that under some circumstances, AceA_Pa_ may play a physiologically relevant role during *P. aeruginosa* propionate catabolism; a role that is normally masked by PrpB_Pa_ functionality.

### Growth of *prpB-*deficient *P. aeruginosa* is inhibited in the presence of propionate

To examine the interplay between PrpB_Pa_ and AceA_Pa_ in more detail, we first generated a *prpB* deletion mutant in PAO1 (a “wild type” *P. aeruginosa* strain used in most laboratories). The Δ*prpB* mutant was unable to grow on propionate as a sole carbon source, but was able to grow in a manner indistinguishable to the WT progenitor on glucose, succinate, or acetate as the sole carbon source (Fig. S3). We next examined growth of the Δ*prpB* mutant (and, as a control, the WT progenitor (PAO1), a Δ*aceA* mutant, and a Δ*prpC* mutant) on a rich medium (LB) in the presence of increasing concentrations of propionate (Fig. 4*C*). To our surprise, the Δ*prpB* was more tolerant of propionate compared with the Δ*prpC* mutant, and grew visibly better than the latter, especially at intermediate concentrations of propionate. This was surprising because we expected the Δ*prpB* mutant to accumulate 2-MIC, which is toxic to the cell. However, the same argument applies to the Δ*prpC* mutant, which would also be expected to accumulate toxic intermediates (principally, propionyl-CoA). We therefore wondered whether the relative growth advantage of the Δ*prpB* mutant was simply a reflection of the differential toxicity of the accumulated intermediates in each mutant, or whether the 2-MICL activity of AceA_Pa_ might help to mitigate toxicity in the Δ*prpB* background. To test this, we made a Δ*prpB* Δ*aceA* double mutant.

### A Δ*prpB* Δ*aceA* double mutant is hypersensitive to propionate

The Δ*prpB* Δ*aceA* double mutant grew normally on succinate, glucose, and LB, but exhibited no growth on either propionate or acetate as a sole carbon source (Fig. S3). In addition, the Δ*prpB* Δ*aceA* mutant exhibited an even greater growth defect than the Δ*prpC* mutant on LB-agar supplemented with exogenous propionate. These data suggested that AceA_Pa_ does indeed help to rescue *P. aeruginosa* from intoxication by self-produced 2-MCC intermediates, and further, that the intermediates which accumulate in the Δ*prpB* mutant are more toxic than those which accumulate in the Δ*prpC* mutant. This “enhanced susceptibility” phenotype could be complemented back to Δ*prpB* levels by expression of *aceA*_*Pa*_ *in trans* in the Δ*prpB* Δ*aceA* double mutant.

To explore the 2-MCC metabolites responsible for the observed propionate-dependent toxicity in the mutants in more detail, we used LC-MS to quantify the 2-MCC pathway intermediates in the WT progenitor (PAO1), the Δ*prpC* mutant, and the Δ*prpB* mutant following addition of propionate (5 mM) to cultures grown in Mops-succinate medium (Fig. 4*D*). After 3 h exposure to propionate, the Δ*prpB* mutant had accumulated approximately 10-fold more 2-MIC than either the WT or the Δ*prpC* mutant. This propionate spiking experiment was also attempted for the Δ*prpB* Δ*aceA* double mutant. However, these cells ceased growth and died so rapidly following propionate exposure that cell pellets could not be obtained for intracellular metabolomics. Instead, we grew the WT and the Δ*prpB* Δ*aceA* double mutant in Mops succinate to an *A*_600_ of 1.0, and then spiked the cultures for just 1 h with 5 mM propionate. The Δ*prpB* Δ*aceA* double mutant accumulated over 1000-fold more 2-MIC and 2-MC compared with the WT (Fig. 4*E*). The intracellular concentration of 2-MIC and 2-MC was comparable in the WT in both the 1 h and 3 h samplings (Fig. 4, *D* and *E*). Together, these data suggest that growth of the Δ*prpB* Δ*aceA* double mutant is rapidly compromised following exposure to propionate, likely due to the accumulation of intracellular 2-MIC and 2-MC.

## Discussion

The 2-MCC is required for infection by many human pathogens and may thus be an important conditionally essential target for antimicrobial development in *P. aeruginosa* (4, 5, 22, 23, 24, 25, 26). However, our understanding of precisely how propionate is metabolized by this organism remains limited (27). Predicting how microbes evolve and adapt upon antimicrobial challenge is notoriously challenging, especially given that these processes are frequently species or even strain specific. Moreover, there is a pervading practice of extrapolating metabolic principles between organisms, in spite of the ever-growing body of evidence suggesting that individual species are often “wired up” very differently relative to the handful of model organisms to which they are often compared (28, 29).

Using a combination of biochemical, structural, and reverse genetics approaches, we have carried out a comprehensive examination of the final step in propionate assimilation by *P. aeruginosa*—catalyzed by the 2-MICL, PrpB. Our data show that PrpB_Pa_ exhibits a strict preference for cleaving 2-MIC, whereas the analogous enzyme in the glyoxylate shunt, AceA_Pa_, is able to catalyze the cleavage of both isocitrate and 2-MIC, albeit, the latter with somewhat lower catalytic efficiency.

The precise residues involved in the selectivity of AceA_Pa_ and PrpB_Pa_ for their respective substrates remains an open question. Simply changing the KRCGH motif of PrpB into the KKCGH motif present in ICLs did not lead to methylisocitrate lyase activity in *E. coli* PrpB (11). A more comprehensive phylogenetic and structural approach hinted that the residues L521 and S523 in *A. fumigatus* PrpB may be critical for substrate selectivity. Mutation of these residues to their counterparts in *A. fumigatus* ICL (L521F, S523T, and the L521F S523T double mutation) yielded proteins that retained methylisocitrate lyase activity, but which also had detectable (albeit, low level) ICL activity (15). However, it is clear that simple substitutions of this type lead to only minor changes in specificity, and that a comprehensive mutagenesis campaign will be required to resolve the issue.

We found that the absence of the *prpB* prevents *P. aeruginosa* from growing on propionate as a sole carbon source. Thus, one would predict that specifically inhibiting PrpB_Pa_ would also lead to rapid cessation of growth on propionate. However, we further show that a *P. aeruginosa* Δ*prpB* mutant is shielded from the toxic impact arising from accumulation of 2-MCC intermediates through substrate promiscuity of the ICL, AceA_Pa_. Surprisingly, in addition to 2-MIC accumulation, we also detected a significant increase in both 2*R*, 3*S*-2MC and 2*S*, 3*S*-2MC in the Δ*prpB* and the Δ*prpB* Δ*aceA* double mutant. These data suggest that the 2-MCC blockage in these mutants leads to a build-up of precursor metabolites beyond those directly upstream of the PrpB_Pa_-catalyzed reaction. This may also contribute to the observed toxicity. The precise cellular targets of 2-MIC or 2-MC (if these are indeed the inhibitory agents) remain to be discovered for *P. aeruginosa*. In spite of extensive efforts, resistance to exogenous propionate exposure could not be selected for in the Δ*prpB* Δ*aceA* double mutant, suggesting that the toxic pathway intermediates likely act through multiple pathways, making the evolution of resistance extremely challenging.

**Figure 5:**
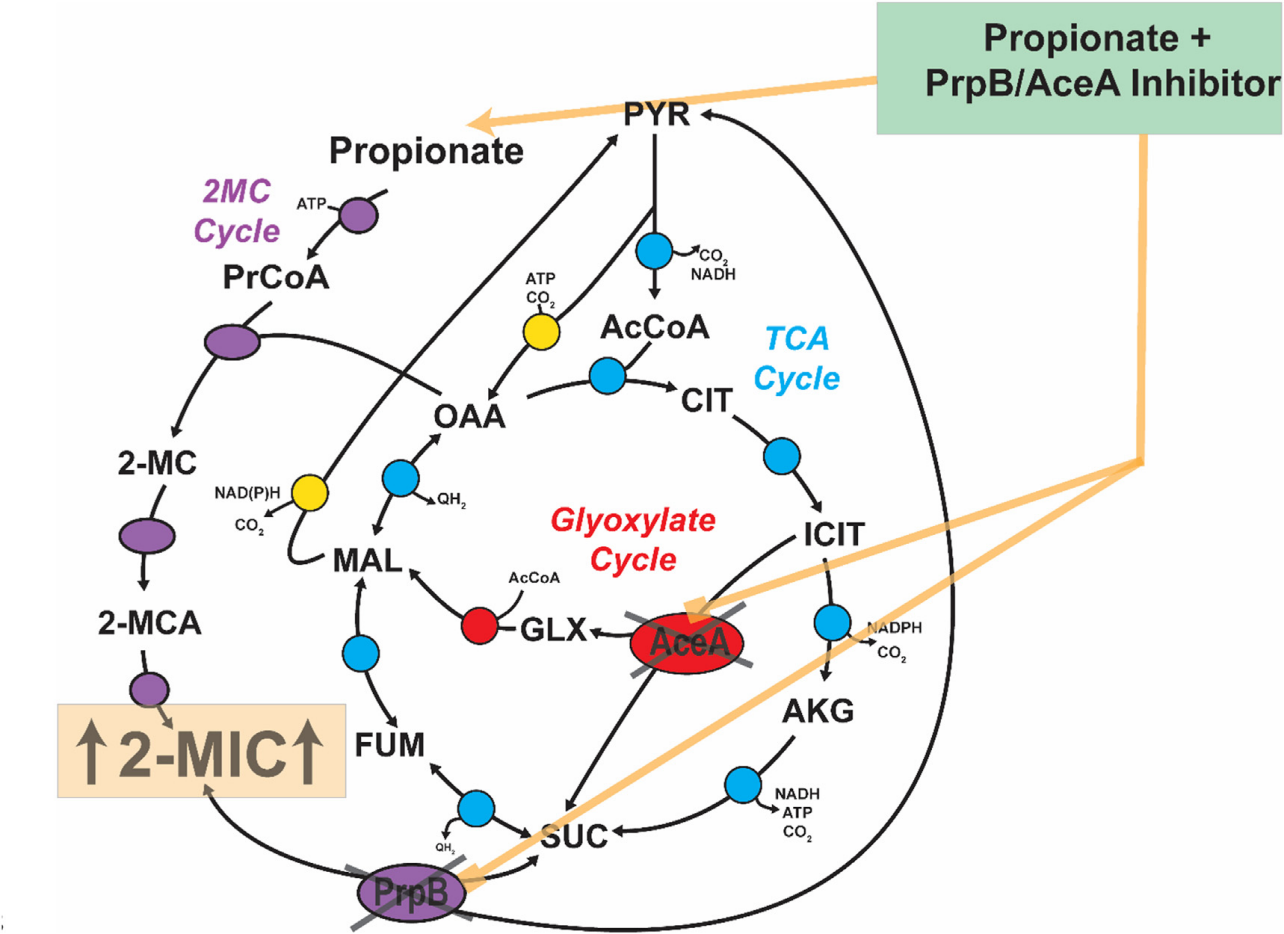
**Summary of the interplay between PrpBPa and AceAPa in the *Pseudomonas aeruginosa* 2-MCC and glyoxylate shunt.** During *P. aeruginosa* growth in the presence of propionate, the 2-MCC is activated and catabolizes the organic acid. In the final step of the 2-MCC, the 2-methylisocitrate lyase, PrpB, cleaves 2-methylisocitrate (2-MIC) to yield pyruvate (PYR) and succinate (SUC). Inhibition of PrpB_Pa_ results in the accumulation of 2-MCC intermediates and subsequent moderate growth inhibition in the presence of propionate. However, the loss of PrpB_Pa_ activity can be partially compensated by the secondary 2-methylisocitrate lyase activity of the glyoxylate shunt enzyme, isocitrate lyase (AceA_Pa_), which presumably lowers 2-MIC (and other toxic intermediates) levels in the cell and maintains viability. AceA_Pa_ is highly expressed during growth on propionate as a sole carbon source. Consequently, a dual-purpose inhibitor, which simultaneously targets both PrpB and AceA could offer potential as an antimicrobial agent that blocks *P. aeruginosa* growth in infection scenarios where short chain fatty acids such as propionate are abundant. 2-MCC, 2-methylcitrate cycle; 2-MIC, 2-methylisocitrate.

Our data show that, through enzyme promiscuity, *P. aeruginosa* encodes a “fail-safe mechanism” which prevents the excessive accumulation of pathway intermediates upon impairment of PrpB_Pa_ function (Fig. 5). It is unclear if this is by “evolutionary design” or is a fortuitous secondary activity. However, it is conceivable that *P. aeruginosa* has bolstered its defenses around this particular 2-MCC reaction, especially considering the immediate consequences of blocking 2-MIC cleavage. Taken together, our data suggest that it may be necessary to target both PrpB_Pa_ and AceA_Pa_ simultaneously if propionate-dependent toxicity is to be developed as an antimicrobial target at this step in the 2-MCC. For example, one obvious mechanism of bypassing PrpB_Pa_ inhibition would be to constitutively activate *aceA*_Pa_ expression (*e.g.*, through mutation of the gene encoding RccR) (21, 30). Consistent with this, and with the known accumulation of propionate in the cystic fibrosis airways, *rccR* loss of function mutations have been detected in *P. aeruginosa* cystic fibrosis isolates (9, 31).

The current work highlights the importance of dissecting pathway interconnections in microbial metabolism, and that microbial metabolism is inherently highly flexible (32). The combination of near-atomic resolution of the PrpB_Pa_ structures described in this work, alongside the existing structural data for *P. aeruginosa* AceA, provides a clear structural template for targeting these key metabolic nodes.

## Experimental procedures

### Growth conditions

*P. aeruginosa* strain PAO1 was routinely grown in lysogeny broth (LB Lennox) (Oxoid Ltd) at 37 °C with shaking at 250 rpm. The strains used in this study are listed in Table S1. Strains were cultured in MOPS media with the relevant carbon sources, as indicated. Cell growth was monitored as absorbance in a spectrophotometer (BioSpectrometer, Eppendorf) at a wavelength of 600 nm (OD_6__00_).

### Cloning, overexpression, and purification

Primers used in this study are listed in Table S2. PrpB_Pa_ and AceA_Pa_ (PA0796 and PA2634, respectively) were PCR-amplified from PAO1 genomic DNA template, and the amplicons were cloned into pET-19m for overexpression. The proteins were overexpressed with a tobacco etch virus (TEV) protease-cleavable N-terminal hexahistidine-tag. Cultures of *E. coli* containing plasmid (pET-19M-*prpB* or pET-19M-*aceA*, as appropriate) were grown at 37 °C (with good aeration at 200 shaking) to an *A*_600_ of 0.6. IPTG was then added to a final concentration of 1 mM to induce protein expression, and the temperature was decreased to 16 °C. After overnight incubation, the cells were harvested by centrifugation (3430*g*, 30 min, 4 °C), and the cell pellets were stored in −80 °C until use. The frozen cell pellets were thawed and resuspended in 20 ml of ice-cold lysis buffer (50 mM Tris–HCl pH 7.5, 300 mM NaCl, 10 mM imidazole, and 5% v/v glycerol). The cells were lysed to completion by sonication on ice. Cell debris was removed by centrifugation (15,000*g*, 30 min, 4 °C), and the supernatant was filtered through a 0.45 μm membrane (Sartorius). The filtered sample was loaded onto a 5 ml Ni-NTA Superflow Cartridge (Qiagen), and PrpB_Pa_ and AceA_Pa_ were purified as previously described (10). The purified samples were dialyzed against 2 × 1 L of storage buffer (50 mM Tris–HCl pH 7.5, 100 mM NaCl, and 5% v/v glycerol) for 16 h at 4 °C. Hexahistidine-tagged TEV protease was added (1:100 ratio of protease:sample) in the dialysis step. The TEV protease and cleaved His_6_ tags were removed by Ni-NTA (Qiagen) chromatography. The purified tag-free proteins were then concentrated by ultrafiltration, flash frozen in liquid nitrogen, and stored at −80 °C.

### Enzyme assays

The 2-MICL activity was measured as follows. Each reaction mixture contained 50 mM Hepes pH 7.5, 2.5 mM MgCl_2_, 5 mM DTT, 1 unit of rabbit muscle LDH (Sigma-Aldrich), 250 μM NADH, and the indicated concentration of *threo*-2-MIC. The *threo*-2-MIC was synthesized in-house and was confirmed by [^1^H] NMR to be a 99% pure racemic mixture of (*2R*, *3S*)- and (*2S*, *3R*)-2-MIC. Before the reaction was initiated, the substrate and buffer were preincubated at 37 °C for 5 min. Reactions were initiated by the addition of PrpB_Pa_ (final concentration of 311 nM unless otherwise stated) or AceA_Pa_ (concentration as indicated) and the A_340_ was monitored using a BioSpectrometer (Eppendorf) for 1 min at 37 °C. Reaction rates were calculated assuming an NADH extinction coefficient of 6200 M^−1^ cm^−1^.

For the inhibitor screening, metabolites were tested at 1 mM final concentration. PrpB_Pa_ was preincubated with each putative regulator for 5 min before the reactions were initiated. Relative activity was measured by comparing the reaction with and without the addition of the putative regulator.

The ICL assay of PrpB_Pa_ and AceA_Pa_ was measured using a phenylhydrazine-based assay. The reaction mixture contained 25 mM imidazole pH 7.0, 10 mM EDTA, 5 mM MgCl_2_, 4 mM phenylhydrazine, and 4 mM DL isocitric acid. Reactions were initiated by the addition of AceA_Pa_ or PrpB_Pa_ at the indicated final concentration, and A_324_ was monitored using BioSpectrometer (Eppendorf) for 1 min.

### Protein crystallization

Crystallization conditions were screened using the sitting drop vapor diffusion technique using a stock solution of protein containing ca. Subsequently, 13 to 15 mg mL^-1^ purified PrpB_Pa_. protein drops were generated using an automated nanoliter liquid handler mosquito HTS (TTP LabTech). Optimization conditions were determined using dragonfly discovery system (TTP LabTech). PrpB_Pa_ crystals were obtained in an 1:1 ratio of protein and reservoir solution (100–200 mM Tris–HCl pH 8.5, 20–30% (w/v) PEG 4000, 100–250 mM LiSO_4_, and 5 mM DTT). Crystals were grown for 9 to 15 days at 19 °C. Crystals were cryoprotected with 25% (v/v) glycerol and 75% (v/v) reservoir solution, mounted in nylon loops (Hampton Research), and flash frozen in liquid nitrogen prior to data collection. For soaking, a solution of pyruvate and succinate (30 mM of both) was added to the crystal-containing drops and left for 3 h prior to mounting.

### X-ray diffraction, structure determination, and refinement

Diffraction data were collected remotely on beamline MX-I03 at the Diamond Light Source Synchrotron. The parameters for the data collection were as follows: wavelength 0.97629 Å, omega (Ω) start: 0°, Ω oscillation: 0.15°, total oscillation: 180°, total images: 1200, exposure time: 0.050 s. Diffraction images were processed using Xia2 DIALS (https://xia2.github.io/using_xia2.html) (33). The structure was determined by MR using Phaser (https://ftp.ccp4.ac.uk/ccp4/6.3.0/unpacked/html/wiki/) (34) with the atomic coordinates of PrpB from *E. coli* (PDB entry: 1MUM) as the search model. Automated refinement was performed using Refmac5 (https://www.ccp4.ac.uk/html/refmac5.html) and PHENIX.refine (https://www.phenix-online.org/documentation/reference/real_space_refine.html) (35). Manual modeling and refinement were performed in COOT (www.jiscmail.ac.uk/cgi-bin/webadmin?A2=COOT;b13b8c75.1804) (36). Data collection and refinement statistics are listed in Table S3. Donor-acceptor distances for hydrogen bonds were in the 3.2–2.2 Å range. The PDB codes for the apo-PrpBPa and PrpB_Pa_/Mg^2+^/pyruvate structures are 6T4V and 6T5M, respectively.

### Analytical ultracentrifugation

Analytical ultracentrifugation-sedimentation velocity was done in the Department of Biochemistry (University of Cambridge) Biophysics Facility. Samples were dialyzed overnight at 4 °C against a buffer solution containing 100 mM NaCl and 50 mM Tris–HCl pH 7.5 to remove traces of glycerol. Data were collected using an AN-60Ti analytical rotor (Beckman Coulter) in a Beckman Optima XL-I ultracentrifuge with absorbance and interference optical detection systems. Protein solution (400 μl volume, concentration approximately 1 mg mL^−1^) and the reference solution (protein-free dialysate) were added to the Epon (epoxy) double-sector centerpieces. All samples were sedimented at 40,000 rpm and 20 °C. Absorbance data (*A*_280_) were collected in intervals of 2 min and interference scans were taken every 1 min. The viscosity and density of the buffer used in the experiments were estimated using SEDNTERP. Data analysis was conducted using SEDFIT (https://sedfitsedphat.github.io/).

### Construction of in-frame *P. aeruginosa* PAO1 deletion mutants

The flanking regions approximately 800 bp upstream and downstream of the desired genes were PCR-amplified (primers in Table S2). The upstream and downstream regions were then overlapped and amplified by PCR. The fragments were then cloned into the suicide vector, pEX19Gm, using Gibson assembly as described previously (10). The resulting deletion plasmid was introduced into *P. aeruginosa* PAO1 by electroporation and transconjugants were selected on LB plates containing 50 μg mL^−1^ gentamicin. Deletion mutants were identified following SacB-mediated sucrose counterselection and confirmed by PCR.

### LC-MS analysis of 2-MCC intermediates

The intracellular accumulation of (2*R*,3*S*)-2-methylcitrate, (2*S*, 3*S*)-2-methylcitrate—the two physiologically occurring diastereomers of methylcitrate—and 2-MIC was measured in cell extracts of the indicated mutant strains using LC-MS as described previously (37, 38). Briefly, cells from 8 ml cultures grown to *A*_600_ = 2 were pelleted and resuspended in 200 μl “supercool” ultrapure water (0 °C) and 1 ml quenching-extraction buffer (95% acetonitrile, 25 mM formic acid, −20 °C). The mixture was vortexed and kept on ice for 10 min, before being clarified by centrifugation at 0 °C. The supernatants were transferred into 3 ml of ultrapure water, before being snap-frozen in liquid nitrogen and lyophilized (Alpha 3–4 LSCbasic, Christ). The freeze-dried samples were diluted in 500 μl precooled resuspension buffer (25 mM ammonium formate, pH 3.0, 2% methanol, 4 °C) and immediately analyzed by LC-MS (QTRAP 6500+ (AB Sciex) coupled to an HPLC system (Agilent Infinity 1290)). Aliquots (5 μl volume) were separated at 25 °C on a C18 column (VisionHT C18 HighLoad, 1.5 μm, 100 × 2 mm) using 0.4% formic acid in ultrapure water as eluent A and a 1:1 mixture of acetonitrile and methanol as eluent B. Flow rate was set to 200 μl min^−1^ with a gradient of A as follows: at 0 min 95%, at 5 min 80%, at 5.5 min 95%, and at 8 min 95%. Detection was performed in multiple reaction monitoring mode with *m/z* of 204.8 (Q1) > 125.0 (Q3) for 2-MC and 2-MIC. MS voltages were optimized for the target compounds. Commercial standards were used for identification, tuning, and quantification.

## Data availability

Coordinates and X-ray crystallographic data for the apo-PrpBPa and PrpB_Pa_/Mg^2+^/pyruvate structures have been deposited in the PDB. The PDB codes are 6T4V and 6T5M, respectively.

## Supporting information

This article contains supporting information (10, 39, 40).

## Conflict of interest

The authors declare that they have no conflicts of interest with the contents of this article.
