# Supplementary material for: The 2-methylcitrate cycle and the glyoxylate shunt in Pseudomonas aeruginosa are linked through enzymatic redundancy
Source: J Biol Chem. 2025 Feb 25;301(4):108355. doi: 10.1016/j.jbc.2025.108355 (PMC11982470; doi:10.1016/j.jbc.2025.108355)
Supplement: Supplementary Information [file mmc1.pdf]

SUPPLEMENTARY FIGURES

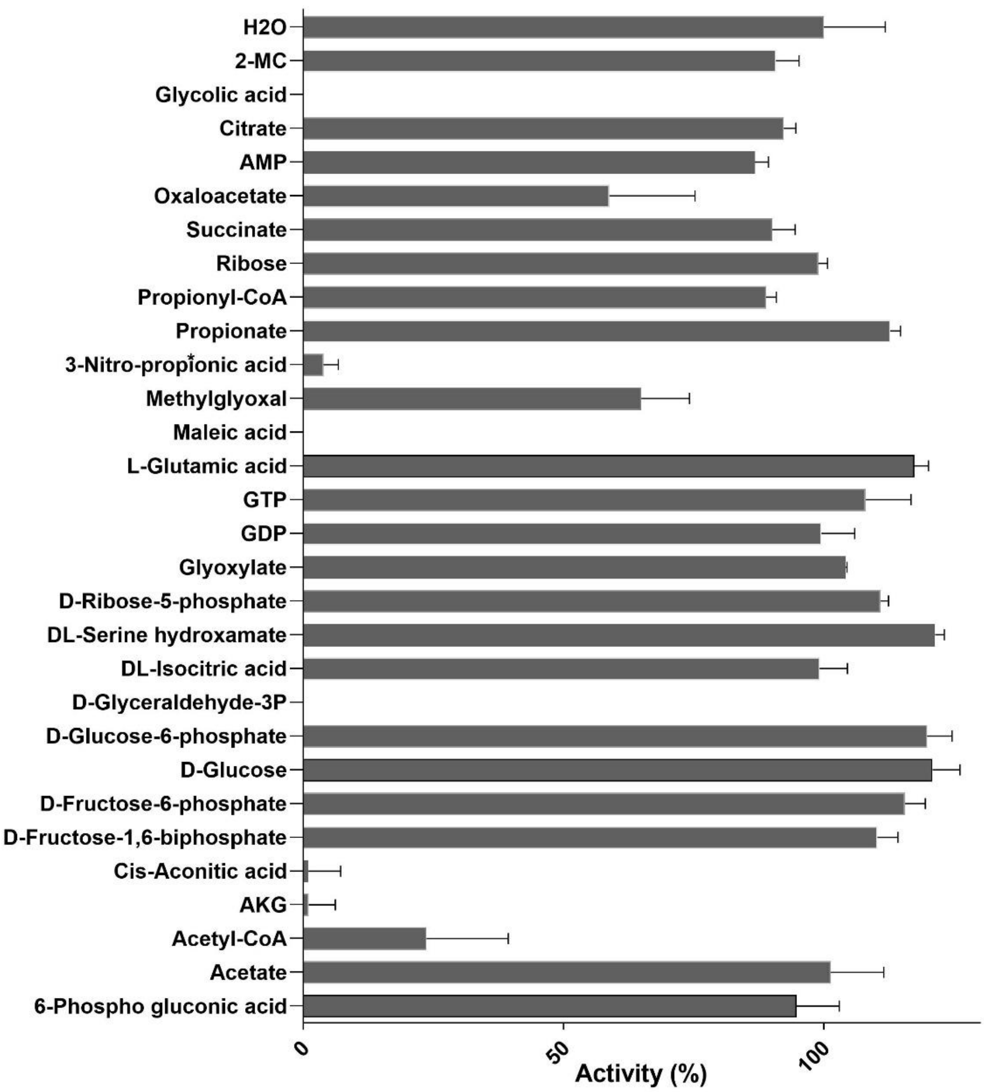

Figure S1. Impact of the indicated metabolites on the 2-methyl*iso*citrate lyase activity of $PrpB_{Pa}$. Each metabolite was tested at 1 mM final concentration. Activity percentage is based on three independent measurements and is shown as mean ± SD relative to the untreated group (H_2O).

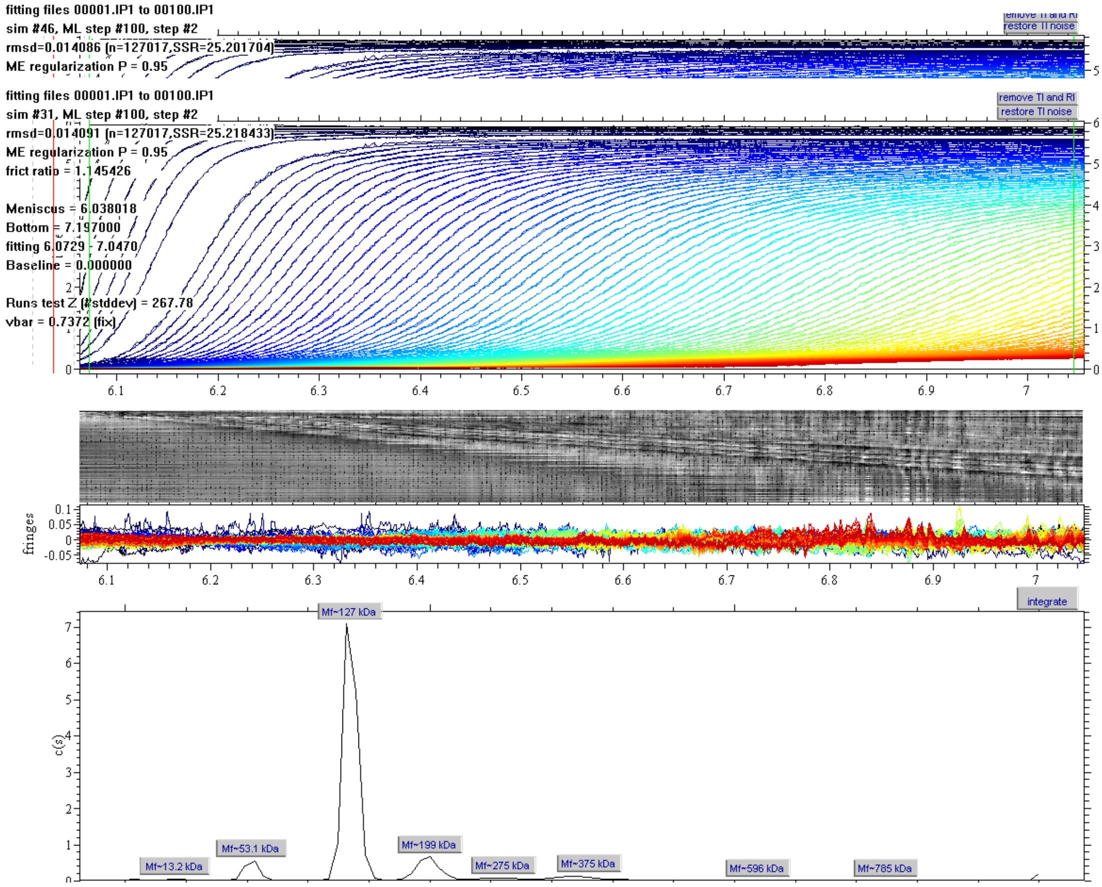

Figure S2. Purified, untagged $PrpB_{Pa}$ behaves as a tetramer in solution. Analytical ultra-centrifugation (AUC) analysis indicates an approximate molecular mass for native $PrpB_{Pa}$ of 127 kDa. For reference, the calculated molecular mass of a single $PrpB_{Pa}$ polypeptide chain is 32.1 kDa.

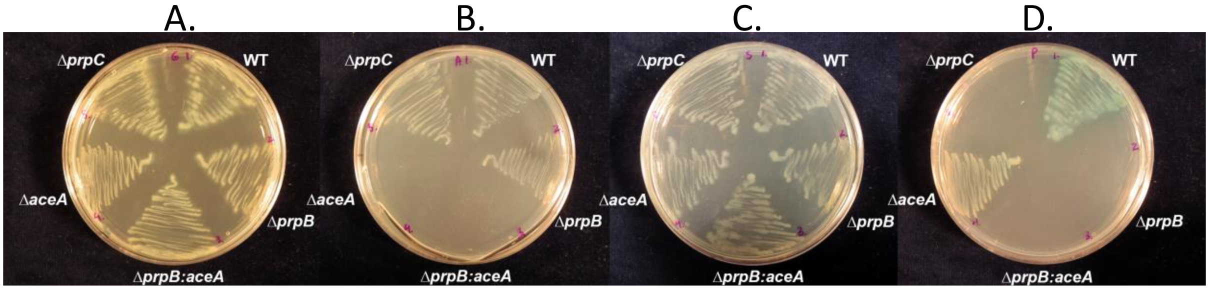

Figure S3. Growth of wild type (WT) *Pseudomonas aeruginosa* (strain PAO1) and the indicated deletion mutants (Δ*prpC*, Δ*aceA*, Δ*prpB*, Δ*prpB* Δ*aceA*) on MOPS minimal agar containing (A) 15 mM glucose, or (B) 40 mM acetate, or (C) 20 mM succinate, or (D) 30 mM propionate as a sole carbon source.

SUPPLEMENTARY TABLES

Table S1: Bacterial strains and plasmids used in this study

**Strain or plasmid**	**Description**	**Source or references**
**Strains**		
E. coli DH5α	*E. coli* strain for cloning	New England Biolabs
P. aeruginosa PAO1	*P. aeruginosa* progenitor strain	(1)
E. coli Rosetta™(DE3)	*E. coli* strain for protein expression	Novagen
PAO1 Δ*prpC*	*prpC* deletion mutant	(2)
PAO1 Δ*aceA*	*aceA* deletion mutant	(2)
PAO1 Δ*prpB*	*prpB* deletion mutant	This study
PAO1 Δ*prpB* Δ*aceA*	Double deletion of *prpB* and *aceA*	This study
**Plasmids**		
pEX19Gm	*P. aeruginosa* suicide vector, Gm	(3)
pET-19m	Protein over-expression vector	
pET-19M:*prpB*	PrpB expression plasmid	This study
pET-19M:*aceA*	ICL expression plasmid	This study

REFERENCES

(1) Stover CK, Pham XQ, Erwin AL, Mizoguchi SD, Warrener P, Hickey MJ, Brinkman FSL, Hufnagle WO, Kowalik DJ, Lagrou M, Garber RL, Goltry L, Tolentino E, Westbrock-Wadman S, Yuan Y, Brody LL, Coulter SN, Folger KR, Kas A, Larbig K, Lim R, Smith K, Spencer D, Wong GK-S, Wu Z, Paulsen IT, Reizer J, Saier MH, Hancock REW, Lory S, Olson M V. 2000. Complete genome sequence of Pseudomonas aeruginosa PAO1, an opportunistic pathogen. Nature 406:959–964.

(2) Dolan SK, Wijaya A, Kohlstedt M, Gläser L, Brear P, Silva-Rocha R, Wittmann C, Welch M. 2022. Systems-Wide Dissection of Organic Acid Assimilation in Pseudomonas aeruginosa Reveals a Novel Path To Underground Metabolism. mBio 13:e02541-22.

(3) Hoang TT, Karkhoff-Schweizer RR, Kutchma AJ, Schweizer HP. 1998. A broad-host-range Flp-FRT recombination system for site-specific excision of chromosomally-located DNA sequences: application for isolation of unmarked Pseudomonas aeruginosa mutants. Gene 212:77–86.

Table S2. Oligonucleotide primers used in this study.

Primer Name	Sequence (5' to 3')
prpB KO UP F	tcggtacccggggatcctctGATGCCCACGAAACTCTC
prpB KO UP R	aatcgatgcgGGTAAGGGACGTCTGGCTC
prpB KO DW F	gtcccttaccCGCATCGATTACCACAGC
prpB KO DW R	catgcctgcaggtcgactctCCCACATGGTCTTGTCGATG
pEX19Gm F	AGAGTCGACCTGCAGGCATG
pEX19Gm R	AGAGGATCCCCGGGTACC
prpB pet19m F NdeI	AAAAAACATATGATGAGCCAGACGTCCCTTAC
prpB pet19m R XhoI	ATCTCGAGTCAGGCGTTCTTCTTCTGCG

Table S3. Crystallographic statistics

Structure	PrpB-Apo	PrpB-Pyruvate & Mg^{2+} bound
**PDB ID Code**	**6T4V**	**6T5M**
**Data Collection**		
Wavelength (Å)	0.97629	0.9686
Resolution range (Å)	64.08-1.807 (1.872-1.807)	37.46-1.76 (1.823-1.76)
Space group	C 1 2 1	C 1 2 1
Unit cell		
a, b, c (Å)	152.859 59.191 148.112	153.49 59.4 147.65
a, b, g (°)	90 120.081 90	90 120.3 90
Total reflections	190287 (27484)	234810 (15956)
Unique reflections	104332 (10378)	97470 (10012)
Multiplicity	1.9 (1.9)	2.4 (2.1)
Completeness (%)	99.10 (99.40)	85.36 (88.52)
Mean I/sigma (I)	6.1 (1.6)	8.5 (1.1)
Wilson B-factor	22.39	27.87
R-merge	0.067 (0.365)	0.095 (0.788)
R-meas	0.089 (0.488)	0.118 (1.003)
R-pim	0.057 (0.320)	0.069 (0.611)
CC1/2	0.994 (0.849)	0.983 (0.454)
**Refinement**		
Resolution range (high resolution) (Å)	64.08 -1.807 (1.872-1.807)	37.46-1.76 (1.823-1.76)
Reflections used in refinement	104257 (10365)	97415 (10009)
Reflections used for R-free	5195 (551)	4805 (485)
R-work	0.1954 (0.2959)	0.2110 (0.3606)
R-free	0.2148 (0.3132)	0.2267 (0.3774)
Number of non-hydrogen atoms	8883	8859
Macromolecules	8445	8412
Ligands	24	28
Solvent	414	419
Protein residues	1106	1105
RMS (bonds) (Å)	0.017	0.015
RMS (angles) (°)	1.76	1.81
Ramachandran favoured (%)	97.89	97.34
Ramachandran allowed (%)	2.11	2.66
Ramachandran outliers (%)	0	0
Rotamer outliers (%)	1.7	1.93
Clash score	2.18	3.38
Average B-factor	30.19	32.4
Macromolecules	30.16	32.47
Ligands	20	32.57
Solvent	31.4	30.9
